# Causality of Biodiversity Loss: Climate, Vegetation, and Urbanization in China and America

**DOI:** 10.3390/s19204499

**Published:** 2019-10-17

**Authors:** Danlu Cai, Klaus Fraedrich, Yanning Guan, Shan Guo, Chunyan Zhang, Leila M.V. Carvalho, Xiuhua Zhu

**Affiliations:** 1 Institute of Remote Sensing and Digital Earth, Chinese Academy of Sciences, Beijing 100101, China; guoshan@irsa.ac.cn (S.G.); zhangcy@radi.ac.cn (C.Z.); 2 Max-Planck-Institute for Meteorology, 20146 Hamburg, Germany; 3 Department of Geography, Earth Research Institute, University of California Santa Barbara, Santa Barbara, CA 93106, USA; leila@eri.ucsb.edu; 4 Center for Earth System and Sustainability, Hamburg University, 20146 Hamburg, Germany; semv097@uni-hamburg.de

**Keywords:** threatened vertebrates, rainfall-runoff chain, vegetation greenness, night-light cities, green and brown cities

## Abstract

Essential for directing conservation resources is to identify threatened vertebrate regions and diagnose the underlying causalities. Through relating vertebrates and threatened vertebrates to the rainfall-runoff chain, to the food chain, and to the human impact of urbanization, the following relationships are noticed: (i) The Earth’s vertebrates generally show increasing abundance and decreasing threatened species indicator (threatened species number/species abundance) for a higher Normalized Difference Vegetation Index (NDVI) or larger city-size. (ii) Regional vertebrates reveal a notable ‘U-shape profile’ (‘step-like jump’) of threatened species indicator occurs in the moderate (high) NDVI regions in China (America). (iii) Positive/green city states emerge in China and are characterized by the lowest threatened species indicators in areas of low to moderate greenness, where the greenness trend of change during the last 30 years is about three times higher in the urbanized areas than over land. (iv) Negative/brown city states emerge in America revealing high threatened species indicators for greenness exceeding NDVI > 0.2, where similar greenness trends are of both urbanized and land areas. The occurrence of green and brown city states suggests a biodiversity change pattern characterized by the threatened species indicator declining from city regimes with high to those with low indicator values for increasing ratio of the city-over-land NDVI trends.

## 1. Introduction

Understanding the ongoing urbanization and climate change effect on biodiversity will help shift traditional management of resources and risks to integrated monitoring and holistic responses across wide ranges of space-time scales [1,2,3]. Biodiversity along altitudinal gradients was previously assumed to increase universally from cool highlands to warm lowlands [4]. However, the interdependence between ecosystems and human activities is hampering the ability to detect universal patterns and the mechanisms determining the distribution of biological diversity on Earth [5]. 

Convincing evidence from the United Nations (UN) confirms that not only is the majority of the world’s population already urbanized, but this level is expected to reach 80% for the developed countries and 60% in the emerging countries by 2050 [6,7]. Venter et al. [8] indicate that 75% of the planet’s land surface is experiencing measurable human pressure, which is widespread and rapidly intensifying in places of high biodiversity. With the increasing availability of high-resolution climate and remote sensing data, broad-scale studies are increasingly likely to estimate magnitude and identify the causality of the global biodiversity loss, which are fundamental for where and how society should be directing conservation resources [2,3].

The idea that climate control on surface energy balance drives the global abundance gradient dates back to the beginning of biogeography [9] and has generated an extensive literature quantifying how biotic and abiotic variables have maintained and driven species’ diversity gradients [10,11,12,13,14]. Among the most important factors, heterogeneities in energy and water availability, linked with the water-energy balance on land surface, explain a major part of the variation in the abundance of a wide range of plant and animal groups [1,13,15]. In addition, the water-energy balance based on a physical model quantifies the impacts of climate change and anthropogenic activities on land surface dynamics [16,17]. This may also explain spatial patterns in the distribution of biodiversity [18] by considering the interdependence of climate forcing, anthropogenic activities, and possible mechanisms underlying their relationships with biodiversity variation. 

China and America contribute similarly to the global overall vertebrate abundance and both countries occupy a similarly large portion of the planet’s land area. However, in the last two decades (1992–2012), the land used for urban construction in China has expanded in all directions from the original core function zone, while no obvious urban area expanding pattern in the main city zone of America could be found [7,19]. 

Vertebrates are participants of an eco-hydrological, natural, and human environment and, therefore, they are embedded in the rainfall-runoff chain, are members of the food chain, and are affected by urbanization. While water and energy fluxes describe the rainfall-runoff chain, species’ abundance as part of the food chain is related to vegetation greenness via the net primary productivity (NPP) (see References [20,21]), and urbanization is related to a country’s state of development. 

Thus, the following questions arise: how do China and America contribute to the global threatened vertebrates, and is it climate-related? Are the numbers of the threatened vertebrates related to urbanization in terms of vegetation greenness changes or city area increases? And is there a threshold (or tipping point) characterizing the relationship between vegetation greenness or city size and the number of threatened species? In this assessment, taxa of three well-known terrestrial vertebrates (mammals, birds and amphibians) are embedded in a state space spanned by balanced water and energy fluxes. Their eco-hydrological states are jointly analyzed with remote sensing-based vegetation greenness and nighttime lighted areas to investigate the mechanisms linking vegetation, vertebrates and urbanization in both a developing and a developed country (China and America), and on a global scale (the world).

## 2. Materials and Methods 

Data on climate, vegetation greenness and urbanization are provided by European Centre for Medium-Range Weather Forecasts ERA-Interim, Global Inventory Monitoring and Modeling System GIMMS Normalized Difference Vegetation Index (1982–2013 averages, [22,23]) and the Defense Meteorological Program Operational Line-Scan System DMSP/OLS nighttime light (2012, [24,25]). The Normalized Difference Vegetation Index (NDVI) is an indicator of the greenness of the biomes (referred to as vegetation greenness hereafter). Taxa of three well-known terrestrial vertebrates (mammals, birds and amphibians) census are available from http://www.biodiversitymapping.org (2010, [3]), which are characterized by their abundance and the threatened species.

### 2.1. Data Preprocessing

The spatial resolutions of the ERA-Interim, GIMMS vegetation greenness and vertebrate datasets are 0.75° by 0.75°, 8 km by 8 km, and 10 km by 10 km, respectively. The spatial area of urbanization obtained from global DMSP/OLS nighttime stable lights products (acquired from National Oceanic and Atmospheric Administration National Geophysical Data Center) with the brightness range digital number DN of 0 to 63 at the spatial resolution of 30 arc-seconds (~1 km) are used. To be comparable, the spatial resolutions are resampled according to the relatively coarse resolution of ERA-Interim into 0.75° by 0.75° by bilinear resampling.

Unlike traditional socio-demographic research using administrative boundaries, population, size or density, and economic indicators to define cities, remote sensing techniques are employed in this study to analyze continuous spatial variation in nighttime light intensity of development or degree of modification. To reduce the effects of over-glow [24,25], only spatially contiguous lighted pixel units (subscript ‘*i*’) with *DN_i_* ≥ 12 are identified as cities (detailed descriptions of data quality control and threshold choosing, see Reference [25]). The area of each pixel within spatially contiguous lighted areas, *a*(*j*), is defined as *a_i_*. Thus, a city (numbered by ‘*j*’) is characterized by its size, *size*(*j*), where, *j* corresponds to the sequence of spatially contiguous areas exceeding the brightness threshold and *i* corresponds to the number of pixels in each of these areas providing the *City size(j) = ∑_i_a_i_(j)*.

### 2.2. Analyses

**Figure 1:**
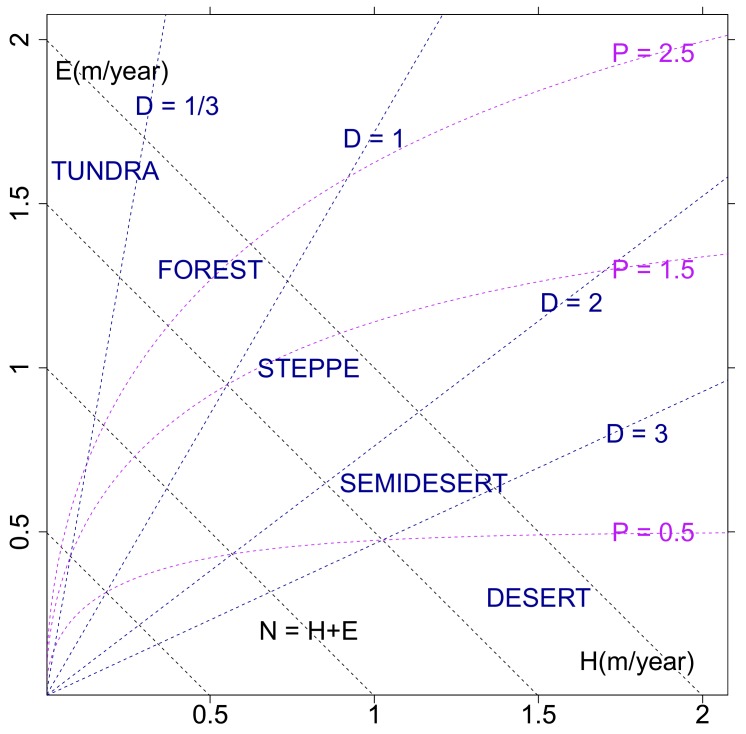
Ecohydrological state space (Stephenson’s diagram) spanned by evapotranspiration and sensible heatflux, *E* and *H*. Net radiation *N*, precipitation *P*, and the dryness ratio, *D* = *N/P*, are included employing the surface energy and water flux balance and the ecohydrological equation of state based on Budyko’s framework (units of fluxes in water equivalents of m/year; see text, Section 2).

**Figure 2:**
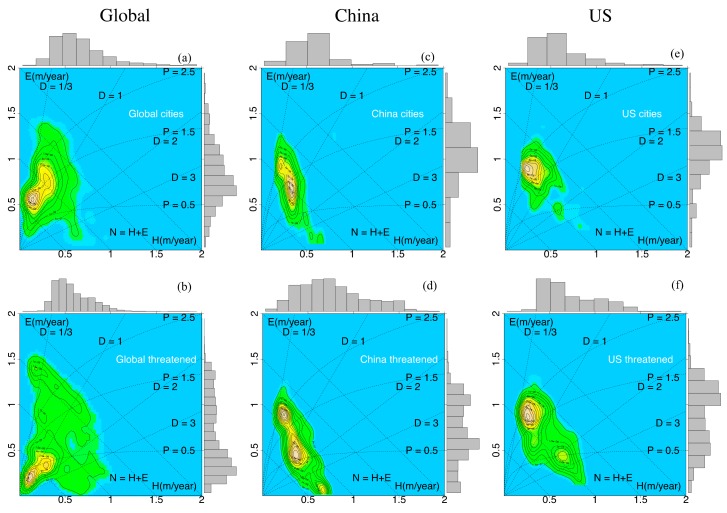
Frequency distribution of the climatological means of surface evapotranspiration *E* and sensible heat flux *H* presented in (*E*, *H*)-state space for (**a**,**b**) Globe, (**c**,**d**) China and (**e**,**f**) America cities (upper row) and threatened species (bottom row).

**Figure 3:**
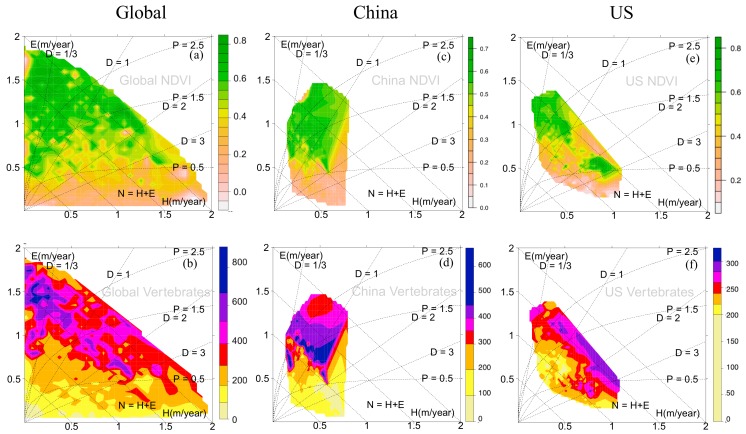
Vegetation greenness (upper row) and vertebrates abundance (bottom row) embedded in (*E*, *H*)-state space across the (**a**,**b**) Globe, (**c**,**d**) China and (**e**,**f**) America (US).

**Figure 4:**
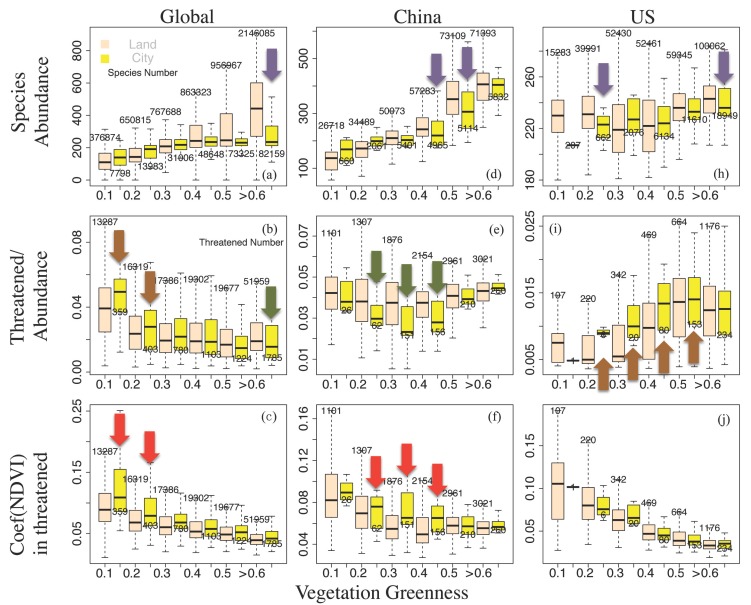
Land-city comparison for across the (**a**–**c**) Globe, (**d**–**f**) China and (**h**–**j**) America: Statistics of the species abundance versus vegetation greenness (upper row), the threatened species indicator (threatened species number/species abundance) versus vegetation greenness (middle row), and the coefficients of annual Normalized Difference Vegetation Index (NDVI) variability versus vegetation greenness (bottom row). Arrows show obvious land and city contrast, and green and brown arrows identify green/brown cities with a positive/negative effect on biodiversity, as quantified by lower/higher threatened species indicator.

**Figure 5:**
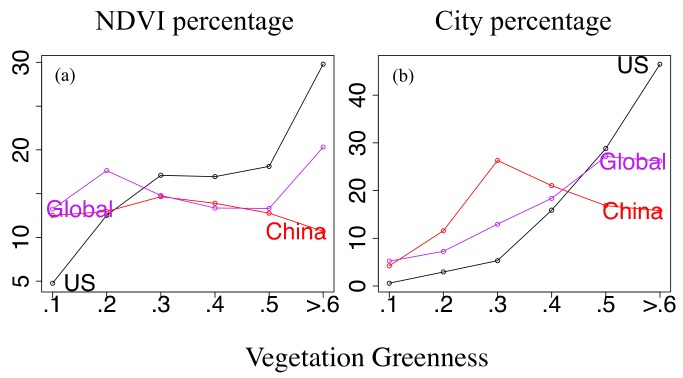
Statistics of (**a**) NDVI percentage (ratio of vegetation covered area (*T* < NDVI < *T* + 0.1)/area), and (**b**) city percentage (fraction of vegetation cover in cities area (*T* < NDVI < *T* + 0.1, light > 12)/area (light > 12)) depending on vegetation greenness for the Globe, China and America.

**Figure 6:**
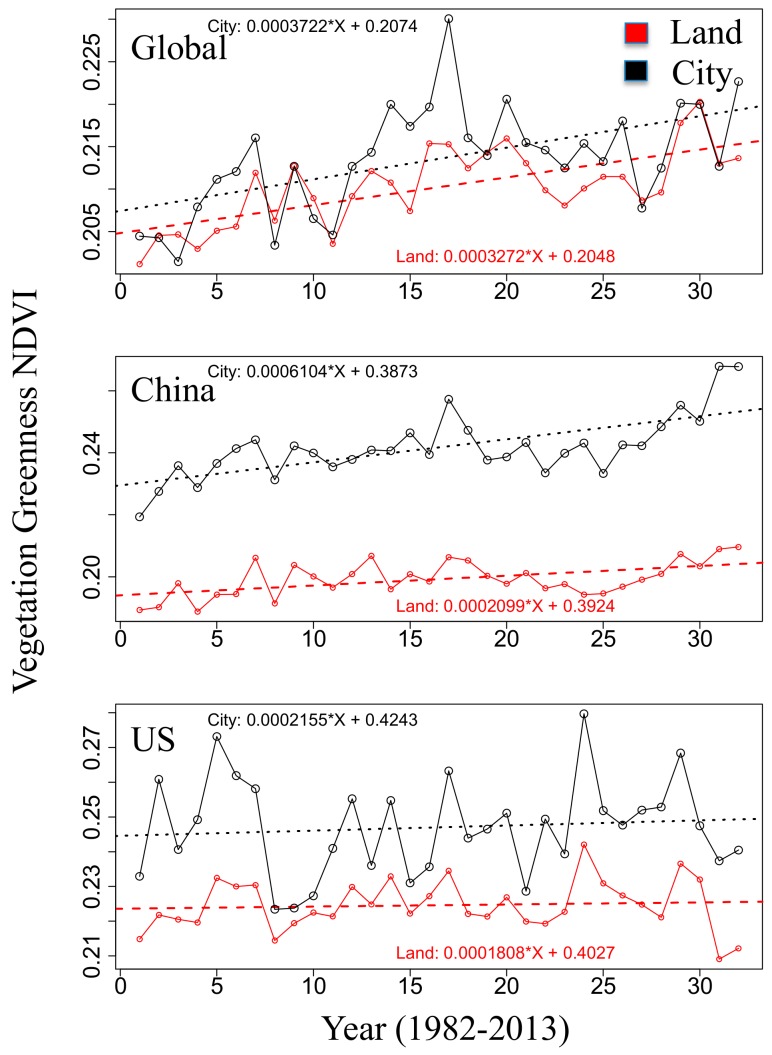
Linear regression statistics of annual averaged vegetation greenness NDVI over land versus within cities for the Globe, China and America: (upper row) global NDVI ranging from 0.1 to 0.3, (middle row) China NDVI from 0.2 to 0.5, and (bottom row) America NDVI from 0.2 to 0.5. Note that the NDVI-intervals selected correspond with the obvious land and city contrast (see arrows in Figure 4).

**Figure 7:**
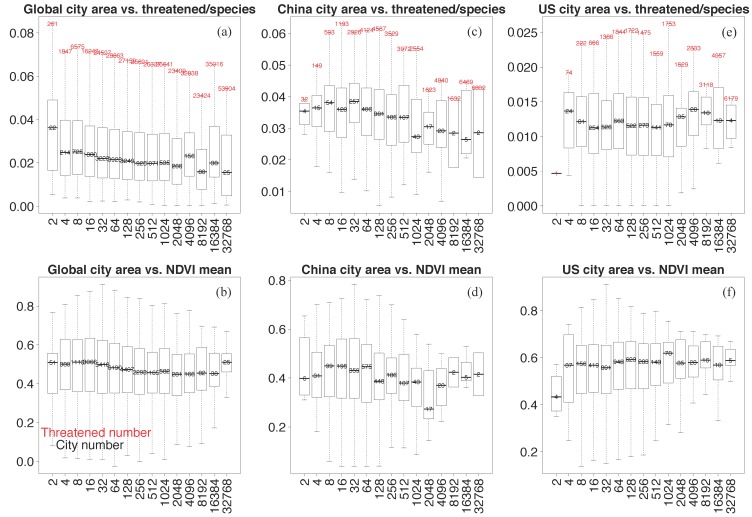
Statistics of city size versus threatened species indicator (upper row) and averaged vegetation greenness (NDVI, bottom row) for the (**a**,**b**) Globe, (**c**,**d**) China and (**e**,**f**) America. City sizes are binned into exponentially wider bins to circumvent the situation that few numbers of cities appear in large city size ranges (city size in km^2^).

First, a snapshot sample of the land coverage of vertebrate taxa for city and non-city conditions provides the statistical setting of the subsequent causality analysis (Table 1), thereby distinguishing China, America and the global environments. Secondly, causality diagnostics commence with an embedding of all variables (vertebrate taxa, vegetation greenness, and cities) describing the climatological setting (in terms of frequency distributions) in eco-hydrological state space (Figure 1, Figure 2 and Figure 3). Finally, the statistics of mutual interrelations, which comprise the greenness dependence of vertebrate taxa and coefficients of NDVI variability (ratio of NDVI standard deviation to climate mean) for city versus land average (city plus non-city), and the city-size dependence of vertebrate taxa and greenness (Figure 4, Figure 5, Figure 6 and Figure 7), provide the basis for the causality analysis.

Eco-hydrological embedding: The interrelation of water and energy supply affecting the eco-hydrological environment is suitably displayed by Stephenson’s [18] diagram, spanned by surface evapotranspiration *E* and sensible heat flux *H* (Figure 1). The surface energy flux balance of the watershed, *N* = *E* + *H*, receives its input from solar radiation, which, if not being stored, is reduced by net outgoing thermal radiation to yield the net radiation flux *N*. Utilized for evapotranspiration *E* and sensible heat flux *H*, the energy balance also provides a constraint on evapotranspiration *E* from land, when all net incoming radiation *N* is used by evapotranspiration *E* and only a negligibly small sensible heat flux *H* remains. In this sense, the maximum possible evapotranspiration *E* is equal to the net radiation *N*, which, defined as potential evapotranspiration [26], characterizes the water demand of a watershed. 

Further relevant fluxes and flux ratios can be included in the (*E*, *H*)-diagram using the energy and water balance equations of the land surface fluxes jointly linked with an equation of state within the Budyko (1974) framework:

(i) The net-radiation as climate forcing, *N* = *E* + *H*, balances the partitioning of the surface latent and sensible heat fluxes and is represented by off-diagonal lines, *E* = *N* − *H*, which characterizes both the water demand and the zonality of continental climates. The Bowen ratios *B* = *H*/*E* are quantified by the slopes of lines (not shown) through the origin (*E* = 0, *H* = *0*).

(ii) Energy and water flux balance at the land surface can be linked by their respective forcing. That is, water demand *N* over water supply or precipitation *P* leads to the dryness ratio (*D* = *N*/*P*), which is introduced to characterize watersheds in terms of how much of the water demand cannot be satisfied by the water supply. Furthermore, the dryness ratio *D* characterizes the geobotanic climate states combining energy and water fluxes by relating water demand (or energy supply) to water supply: Tundra, *D* < *1*/*3*, and forests, *1*/*3* < *D* < *1*, are energy-limited regimes separated from water-limited climates types like Steppe and Savanna, *1* < *D* < *2*, semi-desert *2* < *D* < *3*, and desert *3* < *D* [26]. 

(iii) In addition to the surface energy and water flux balance equations, further information is obtained employing the Budyko framework [26] by introducing, for example, Schreiber’s [27] equation of state, *E*/*P* = *1* – *exp*(−*D*). Representing the climate mean rainfall-runoff chain on watershed scale, it can be theoretically derived employing a stochastic Ansatz as in thermodynamics [28] or a suitable interpretation of the Legendre Transformation [29]. Thus, precipitation *P* and dryness ratio *D* can also be visualized in Stephenson’s diagram (Figure 1) by *H = P ln*(*1 – E/P*) *^−1^*– *E* and *E = H* (*1* – *exp*(*−D*))/(*D – 1* + *exp*(−*D*)) and visualized as isolines in (*E*,*H*)-space. 

Note that ERA-Interim energy fluxes are provided as half-day integrals (*J* m^−2^ (12 h) ^−1^ or 2(365) *J* m^−2^ yr^−1^) and converted to water equivalents of 10^3^ kg m^−2^ yr^−1^ (or m yr^−1^) using the latent heat for condensation of water, *L* = 0.25 10^7^*J* kg^−1^ at 0 °C. Thus, an energy flux of 1 *J* m^−2^ (12 h) ^−1^ yields a water flux equivalent of 2.920 × 10^−7^m × yr^−1^. 

Vegetation greenness and city size: Vegetation greenness-dependent conditional distributions are presented by stepwise increasing NDVI values on the globe, in China and America for (a) species abundance, (b) threatened species indicator (threatened species number/species abundance), and (c) coefficients of year-to-year vegetation greenness variation (ratio of NDVI standard deviation to its climate mean, referred to coefficients of NDVI variability hereafter). These distributions are then extended by comparing with the fractional area coverage of vegetation and urbanization and linear greenness trends of about the last thirty years. Finally, the city size spectrum analysis [7] inspired by the Zipf’s power-law is displayed to diagnose the relationship among city size, vegetation greenness and the threatened species indicator. It implies that small occurrences are extremely common whereas large instances are extremely rare [30]. As small sized cities prevail in number compared with megacities, the city size is sampled in exponentially wider bins with a base of 2 km^2^.

## 3. Results and Discussion

Table 1 provides the statistics of three vertebrate abundances and their threatened species taxa within and outside of the cities of China, America and the globe: 

(i) China and America contribute similarly (5.9% and 5.4%) to the global overall vertebrate abundance and both countries occupy ~6% each of the planet’s land. However, with a slightly higher percentage of overall vertebrate abundance, China accounts for 9.6% of the globally threatened species (145,196) compared with 2.1% in America (Table 1a–c). 

(ii) City areas (Table 1d) cover 6.0% (12.3%) of the landmass of China (America). Countrywide, the threatened species percentage within cities is expected to be proportional to the city area cover if cities show no extra effects than their land average, but several taxa show higher percentages of threatened species ratio within cities than the corresponding city percentage, particularly for America, indicating 16.1% (22.3%) threatened birds (mammal), which is greater than 12.3% in city areas cover of the American landmass.

(iii) Statistics based on DMSP/OLS nighttime stable lights products show the urban construction changed over time in Chinese (American) cities, expanding from 1.3% to 6.0% (10.7% to 12.3%) during 1992 to 2012 [7], meanwhile China accounts for ~4 times more globally threatened species than America (9.6% versus 2.1%). However, relatively high percentages of threatened vertebrates compared with their city percentage indicate extra negative effects in American cities than non-cities. While in China, the threatened species ratios for cities show percentage values similar to (or lower than) the relative city area cover.

Therefore, it is reasonable to hypothesize that the expansion of human land use associated with Chinese cities over the last decades is not suitable for explaining that urbanization may or will threaten more species. The underlying reasons comprise three components of three mutual relationships, which are analyzed in the following three subsections: The ecohydrological climate and city embedding (through the water and energy balance, Section 3.1), land cover and city connection (by vegetation greenness, Section 3.2) and, finally, the city size causalities (Section 3.3).

### 3.1. Eco-Hydrological Embedding

The latest fine-scale biodiversity data illustrate the patchy and non-uniform geographical distribution of global species and threatened species [3]. For amphibians, birds and mammals, the greatest diversity areas occur in nearly similar locations, concentrating in the moist forests of the Amazon, Brazilian Atlantic Forest, Congo, Eastern Arc of Africa, and the Southeast Asian mainland and islands. Abundance patterns of threatened species differ dramatically from those of overall abundance: threatened amphibians are globally scattered and occupy in total only a tiny fraction of the global land area, threatened birds concentrate in the Andes, southeast Brazil, and Southeast Asian islands, whereas threatened mammals are concentrated on the mainland and islands of Southeast Asia (for more details, see Reference [3]). 

Measures of urbanization, vertebrate species and vegetation are jointly embedded in an eco-hydrological evapotranspiration and sensible heat flux (*E, H*)-space (Figure 2 and Figure 3).

(i) Urbanization: The frequency (Figure 2, upper row, number density) distributions of Global, Chinese and American cities are characterized by unimodal distributions with maxima below the separation of energy-limited from water-limited regimes, *D ~* 1, where the tropical and temperate forests are concentrated (see biome-dryness analysis, [31]). While American cities are mainly concentrated in the domains of net radiation 1 < *N* < 1.5 m/yr and precipitation 1.5 < *P* < 2.5 m/yr, the cities in China and also globally occupy the climate regime of net radiation 0.5 < *N* < 1.5 m/yr and precipitation 0.5 < *P* < 2.5 m/yr.

(ii) Threatened vertebrates (Figure 2, bottom row): Global threatened vertebrates are concentrated in the domains of net radiation *N* < 1 m/yr and along a constant dryness line *D* ~ 1, while in China and America, threatened vertebrates are scattered. In China, the frequency peaks of threatened vertebrates are separated by dryness values of *D* < ~1, ~2 and *D* > 3. In America, threatened vertebrates show two modes, one in the energy-limited regime (*D* < 1), coinciding with the peak of the American city distribution (Figure 2e), and the other in the water-limited regime (*D* > 1). This is in agreement with the statistics summarized in Table 1d, showing a relatively high percentage of threatened vertebrates in American cities compared with their city percentage.

(iii) Vegetation greenness and vertebrate abundance (smoothed frequencies, Figure 3) extend Stephenson’s [18] (*E, H*)-diagram designed to embed vertebrate societies. High-vegetation greenness is mainly located in energy-limited regions of low dryness *D* < ~1, where vertebrates also show high abundance. Globally, and for America (Figure 3b,f), the abundance pattern increases along lines of constant dryness, which is in agreement with plant distributions [18] and extends from cold desert shrub and short grass prairie, via tall grass prairie to deciduous forests. In China (Figure 3d), the line of constant sensible heat flux *H* represents a transection of increasing abundance, which corresponds to the geographical distribution from the northwest to the southeast of China.

### 3.2. Urbanization, Vegetation, and Vertebrates

The following general global patterns (Figure 4a–c) emerge for the stepwise increasing vegetation greenness: Global vertebrate abundances increase, the threatened species indicators decrease, and the coefficients of NDVI variability also decrease. The reduced variability of vegetation greenness being related with enhanced species diversity has been described [32], as it is supported here showing high NDVI values being linked with low coefficients of NDVI variability and with low relative numbers of threatened species. As the general global pattern, for the stepwise increasing vegetation greenness, the increased species abundance and the reduced coefficients of NDVI variability are also observed in America and in China, where no obvious regional tendency differences could be found in the Figure 4 upper and bottom rows. 

Although non-city regions or land-averaged cases may include geographical locations with presumably a poor animal population, land-city differences in vertebrate abundance (purple arrows in Figure 4, upper row) show higher value over land in the high NDVI areas. It may be simply attributed to the high NDVI areas being affected by urbanization. For the threatened species analyses, this area/population effect needs to be reduced or eliminated by employing the threatened species indicator (threatened species number/species abundance, middle row). Unlike the general global pattern, those threatened species indicators (Figure 4 middle row) show regional and land versus city discrepancies associating with positive/negative city effects as follows: 

Threatened species indicators: The general global pattern shows a higher (lower) threatened species indicator in low (high) NDVI regions for cities compared with the land (Figure 4b). China cities (compared to land) show a deeper ‘U-shape profile’ with a lower threatened species indicator occurring in an interval of moderate greenness, 0.2 < NDVI < 0.5 (Figure 4e), while in America, the threatened species indicator for cities rises with a ‘step-like jump’ when greenness exceeds the NDVI threshold of ~0.2 (compared with ~0.4 for land, Figure 4i). 

The lower/higher threatened species indicators (green/brown arrows, Figure 4) characterize cities with a positive/negative effect for species conservation. Globally, lower greenness values of 0.1 < NDVI < 0.3 show a negative city effect, for which the coefficient of annual NDVI-variability is higher in cities than over land (red arrows, Figure 4). However, cities in China and America, which are covered by moderate greenness values of 0.3 < NDVI < 0.5, suggest a positive (negative) influence on biodiversity linked with the coefficient of NDVI-variability being higher (constant) within cities. That is, high coefficients of annual NDVI-variability in cities do not necessarily suggest a negative city effect for species conservation.

As the city cover in China is still low (~6% of China landmass), it cannot be excluded that there may be a threshold value of relative city area coverage, beyond which the ratio of threatened species for cities increases. To clarify that, we compare the relative (percentage) city cover changes in China and America for stepwise increasing vegetation greenness (Figure 5): Highest city coverage occurs in China at NDVI ~ 0.3, while coverage of American cities exceeds that of cities in China for NDVI > 0.4. Comparing this with the threatened species indicators in Figure 4e,i, we note that America shows a negative city effect, while China reveals a positive city effect even when city occupation is highest at NDVI ~ 0.3.

Urbanization and nature conservation: On a global scale and in America, a higher percentage of the total land area (the relative pixels frequency) is covered by vegetation of higher greenness, while in China, the vegetation cover shows an almost uniform frequency distribution (~13%, Figure 5a). Meanwhile, on a global scale and for America, the percentage of total city area occupied by vegetation (Figure 5b) increases with increasing greenness, while it is unimodal for cities in China (that is increasing for NDVI < 0.3 and decreasing for NDVI > 0.3). That is, the positive city effect in Figure 4e overlays with the unimodal peak and is more likely to occur in China with its large moderate greenness coverage and the large occupation of these regions of moderate greenness by cities.

Associated with the ‘step-like jump’ of the threatened species indicator in American cities (the negative city effect commencing with NDVI 0.2-threshold, particularly for NDVI 0.4-threshold, Figure 4i), nature conservation becomes relevant ranging from dense human settlement cover to highest concentration of urbanization of America (Figure 5b). Compared with cities in China, the highest concentration of American urbanization occurs in regions where vegetation greenness can hardly be enhanced.

The observed positive/negative city effect for species conservation is explained by the vegetation greenness trend over the past thirty years (Figure 6). For global (American) cities with greenness of about 0.1 < NDVI < 0.3 (0.2 < NDVI < 0.5), the vegetation greenness trends are comparable with the trends observed over land and occur jointly with an increased (similar) coefficient of NDVI-variability. In this sense, cities, which are characterized both by an equal or larger coefficient of NDVI-variability and by a similar vegetation greenness trend, may impose a negative effect on species conservation.

However, cities in China (Figure 6), which are characterized by a low to moderate greenness cover, 0.2 < NDVI < 0.5, show an upward trend of vegetation greenness during the last thirty years, which is three times as large as over land. It is linked with a coefficient of NDVI-variability being larger for cities than over land. In this sense, cities, which are characterized by increased coefficients of NDVI-variability and associated with overwhelmingly positive vegetation greenness trends (larger than over land), can be suitably defined as being governed by a positive city effect for species conservation.

This positive effect of (green) cities in China, in contrast to the negative effect of (brown) cities in America, is in agreement with Kraas et al. [33]. That is, the habitats in many of the green cities provide the basis for a surprisingly high level of biodiversity, whose value is often underestimated, as it is in contrast with the idea that city expansion into the surrounding areas and the creation of new settlements destroys near-natural ecosystems and their biodiversity.

### 3.3. City Size Relation

Cities of a larger city size are likely to show a threatened species indicator reduction (Figure 7a,c) which is subject to small fluctuations (e.g., Figure 7e, America megacities ranging from 2048 to 8192 km^2^). The general relation of city size with mean vegetation greenness explains that a larger city size is linked with less annual greenness variability (Figure 7 bottom row, see vertical spread in boxplot), leading to a decreasing number of threatened species indicators. Fluctuations of the threatened species indicator in America may be related to urbanization with a dense human settlement in Figure 5b.

In summary, threatened species are far more abundant in China than in America, but the percentage of threatened species related to the relative city cover is less for cities in China than in America, despite the fact that cities in China have been largely expanding in the last decades. Therefore, the human land use expansion by urbanization in China does not support the explanation that cities may or will threaten more vertebrates. Instead, the threatened vertebrates are affected by the interrelationship of the rainfall-runoff chain, the food chain in terms of vegetation greenness NDVI, and the human environment. More specifically, the rainfall-runoff chain of the (*E*, *H*)-state space (Stephenson’s diagram) for embedding all variables (vertebrate taxa, vegetation greenness, and cities) in America indicate that, the most condensed peak of the threatened vertebrates coincides with the peak of the American city distribution. In addition, observed positive/negative city effects emerge under moderate NDVI conditions which, due to the greening of cities (about three times larger compared to the total land), leads to the threatened species indicators being smaller (see urbanized China, Figure 4e and Figure 6, middle row). In contrast, cities of America compete with vertebrates of high abundance (Figure 4i) over environments whose greenness is high, and because greenness values are relatively high in both urbanized and non-urban regions of America, their greenness growth is limited and of similar magnitude (Figure 6, bottom row).

## 4. Conclusions

Facing the foreseeable population/urbanization growth being in conflict with conservational protection, it is essential to identify priority regions where biodiversity (suitably measured by vertebrate species abundance) is under threat due to human land use. Fundamentally, we must know where human land use by urbanization is critical, how human actions in cities threaten certain species, and what generates the heterogeneities in threatened species on a global and a countrywide scale. Therefore, a pixel scale-based vegetation greenness analysis and city scale-based causality interpretation have been employed and provide the following insights:

(i) A general pattern describing the vegetation greenness (NDVI)-dependent vertebrate abundance emerges globally, and countrywide for China and America: With increasing NDVI, vertebrate abundance increases and the coefficients of NDVI variability decreases, while vertebrate abundances are not uniform in different geographical ranges [34]. For cities in China and on a global scale, but not in America, a general pattern of city size dependence emerges: Enhanced city sizes are associated with reduced vegetation greenness variability and reduced threatened species indicator.

(ii) Compared with this global setting and on the geographical scales of China and America, the threatened species indicator (threatened species number/species abundance) reveals unique distributions. Globally, for both city and non-city environments, a low threatened species indicator is associated with larger NDVI values. However, China shows a ‘U-shape profile’ of the threatened species indicator occurring in the moderate NDVI regime, whereas America reveals a notable ‘step-like jump’ with the threatened species indicator increasing towards regions of higher NDVI. Thus, the threatened species indicator appears to be a sensitive and vulnerable parameter characterizing city versus non-city regimes, whereas both NDVI and the coefficients of year-to-year NDVI variability do not provide a consistent measure of the city versus non-city greenness.

(iii) The land-city comparison shows that, in the global mean, a higher (lower) threatened species indicator is connected with low (high) NDVI regions. However, cities (compared to land average) in China show a deeper ‘U-shape profile’ with lowest values of the threatened species indicators in areas of low to moderate greenness, 0.2 < NDVI < 0.5. In cities of America, a ‘step-like jump’ from low to high threatened species indicators occurs at NDVI exceeding ~0.2 (compared with land average ‘jump’ observed for NDVI exceeding ~0.4). That is, the expansion of cities into the surrounding regions and the creation of new settlements, such as in China, does not necessarily destroy near-natural ecosystems reducing their biodiversity. Thus, lower/higher values of threatened species indicators are useful measures to identify a positive/negative city effect for species conservation which, in addition, is also connected with the vegetation greenness and with its trend over the last thirty years.

(iv) Cities in China reveal a positive city effect: They occupy an area of low to moderate greenness with potential to enhance, and reveal a trend of vegetation greenness increasing during the last 30 years, which is about three times larger for cities than land, although they are linked with an increased coefficient of NDVI-variability. A negative effect of global (American) cities occurs where both cities and land occupy an area of similar lower (moderate) greenness linked with a larger (unchanged) coefficient of NDVI-variability but share similar vegetation greenness trends of cities and land. 

Thus, for species conservation, city percentage distribution associated with a positive/negative city effect (green and brown cities) identifies the effect of how urbanization generates the heterogeneities in threatened species on a global and a countrywide scale. The underlying reason is associated with a biodiversity change pattern characterizing the threatened species indicator declining from a city regime with a high indicator value to one with a low indicator value, when increasing the fractional city-over-land NDVI trend in moderate NDVI regions. Therefore, for natural conservation, we suggest it is beneficial for a city to provide vegetation of higher greenness compared with their land, including (i) to increase vegetation greenness in cities of moderate NDVI, and (ii) to confine the expansion of cities into surrounding areas of high NDVI.

## Figures and Tables

**Table 1:** Urbanization (representative year 2012) and vertebrates (representative year 2010): China and America (USA) compared with the globe in percentage of (**a**) area coverage, (**b**) vertebrate abundance and (**c**) threatened vertebrates, and (**d**) relative city nighttime lighted area and the percentage of threatened species inside China, America and the globe.

	China (%)	USA (%)	Global
a) Total Land Area	**6.3**	**6.1**	**148,940,000 km^2^**
b) Vertebrates Abundance	**5.9**	**5.4**	**5,930,794 number**
Amphibian Birds Mammal	4.4 5.9 6.1	7.3 5.2 5.8	283,256 4,376,655 1,270,883
c) Threatened Vertebrates	**9.6**	**2.1**	**145,187 number**
Amphibian Birds Mammal	29.5 7.5 12.2	5.3 2.7 0.8	2,892 92,513 49,782
d) Cities*/Non-Cities in Continental/Global Land	**6.0/94.0**	**12.3/87.7**	**4.5/95.5%**
Threatened Amphibian in/Out Cities Threatened Birds in/Out Cities Threatened Mammal in/Out Cities	7.0/93.0 8.0/92.0 4.4/95.6	9.8/90.2 16.1/83.9 22.3/77.7	7.8/92.2 4.3/95.7 3.3/96.7

*cities = exceeding night light threshold DN > 12. a, b and c in percent of global reference, d, in percent of local reference. red means threatened indicator in cities greater than the city percentage
